# Integrated fecal microbiome–metabolome signatures reflect stress and serotonin metabolism in irritable bowel syndrome

**DOI:** 10.1080/19490976.2022.2063016

**Published:** 2022-04-21

**Authors:** Zlatan Mujagic, Melpomeni Kasapi, Daisy MAE Jonkers, Isabel Garcia-Perez, Lisa Vork, Zsa Zsa R.M. Weerts, Jose Ivan Serrano-Contreras, Alexandra Zhernakova, Alexander Kurilshikov, Jamie Scotcher, Elaine Holmes, Cisca Wijmenga, Daniel Keszthelyi, Jeremy K Nicholson, Joram M Posma, Ad AM Masclee

**Affiliations:** a Division Gastroenterology-Hepatology, Maastricht University Medical Center+, Maastricht, The Netherlands; b Nutrim School for Nutrition and Translational Research in Metabolism, Maastricht University, Maastricht, The Netherlands; c Division of Systems Medicine, Department of Metabolism, Digestion and Reproduction, Faculty of Medicine, South Kensington Campus, Imperial College London, London, UK; d Division of Digestive Diseases, Department of Metabolism, Digestion and Reproduction, Faculty of Medicine, Hammersmith Campus, Imperial College London, London, UK; e Department of Genetics, University of Groningen, University Medical Center Groningen, Groningen, The Netherlands; f The Australian National Phenome Center, Harry Perkins Institute, Murdoch University, Perth, Australia

**Keywords:** Irritable bowel syndrome, gut microbiota, microbiome, gut metabolome, fecal metabolome, stress, serotonin, gut-brain, host-microbiome interaction

## Abstract

To gain insight into the complex microbiome-gut-brain axis in irritable bowel syndrome (IBS), several modalities of biological and clinical data must be combined. We aimed to identify profiles of fecal microbiota and metabolites associated with IBS and to delineate specific phenotypes of IBS that represent potential pathophysiological mechanisms. Fecal metabolites were measured using proton nuclear magnetic resonance (^1^H-NMR) spectroscopy and gut microbiome using shotgun metagenomic sequencing (MGS) in a combined dataset of 142 IBS patients and 120 healthy controls (HCs) with extensive clinical, biological and phenotype information. Data were analyzed using support vector classification and regression and kernel t-SNE. Microbiome and metabolome profiles could distinguish IBS and HC with an area-under-the-receiver-operator-curve of 77.3% and 79.5%, respectively, but this could be improved by combining microbiota and metabolites to 83.6%. No significant differences in predictive ability of the microbiome–metabolome data were observed between the three classical, stool pattern-based, IBS subtypes. However, unsupervised clustering showed distinct subsets of IBS patients based on fecal microbiome–metabolome data. These clusters could be related plasma levels of serotonin and its metabolite 5-hydroxyindoleacetate, effects of psychological stress on gastrointestinal (GI) symptoms, onset of IBS after stressful events, medical history of previous abdominal surgery, dietary caloric intake and IBS symptom duration. Furthermore, pathways in metabolic reaction networks were integrated with microbiota data, that reflect the host-microbiome interactions in IBS. The identified microbiome–metabolome signatures for IBS, associated with altered serotonin metabolism and unfavorable stress response related to GI symptoms, support the microbiota-gut-brain link in the pathogenesis of IBS.

## Introduction

The gastrointestinal (GI) tract and its microbiome is a potent, but incompletely understood, metabolic organ system, with capacities reaching beyond the primary function of nutrient processing and absorption,^1^ including the regulation of complex (neuro)endocrine and immune pathways.^2–4^ Dysregulation of these processes is presumed to play a key role in the development of several GI and extra-intestinal disorders.^5–7^ Among these, the prevalent GI disorder irritable bowel syndrome (IBS) is prototypical for involvement of the microbiome-gut-brain axis.^7–9^ The complexity of potential etiological factors in IBS, involvement of psychological comorbidity and its heterogeneity have proven to be a hurdle in the pursuit of biomarkers and the development of more efficacious treatment strategies.^8,10,11^

Previously, it was shown that clear differences exist between gut microbiota profiles of patients with IBS, as compared to subjects with other GI disorders and healthy controls (HCs).^12–14^ Nevertheless, consensus on the exact nature of changes in gut microbiota composition in IBS is lacking.^15^ Furthermore, next to the microbial composition, the available data on the microbial metabolic activity is limited and inconclusive,^13,16–18^ while the host–microbiome interaction may be largely driven by the metabolic microbial processes. In addition, in particular the knowledge regarding how changes in gut microbiome and metabolome in IBS reflect on potential pathophysiological mechanisms or subgroups of IBS patients, in this heterogeneous disorder is very limited, and studies assessing these pathways are needed to improve our understanding of host–microbiome interaction in IBS. Intraluminal metabolic patterns in the gut are complex and are modulated by various factors such as microbial activity, diet, medication use and host GI function and metabolism. We previously have studied several of these mechanisms separately in different sub-populations of the Maastricht IBS (MIBS) cohort.^11,12,19–22^ However, to get insight into the host–microbiome interaction in IBS, all these factors must be taken into account together when studying gut microbiota composition and metabolic activity.

In the current study, we therefore combined analyses of gut microbiota composition and its metabolic capacity using whole-genome shotgun metagenomic sequencing (MGS) and with novel data on gut microbiota metabolic activity using proton nuclear magnetic resonance (^1^H-NMR) spectroscopy, in a deeply phenotyped cohort of primary to hospital care IBS patients with a control group of healthy individuals. The extensive metadata available in this cohort comprises GI and psychological symptoms, diet, medication use, comorbidity, intestinal permeability, visceral hypersensitivity and multiple plasma and fecal biomarkers related to inflammation and neurohormonal activity.

The objectives were to 1) identify profiles of fecal microbiome and metabolome associated with IBS, 2) study associations of fecal microbiota composition, its metabolic potential and activity with specific phenotypes of IBS and 3) study the host–microbiome interaction using metabolic reaction networks integrated with microbiome information and pathway analyses.

## Results

Fecal samples were available for 314 individuals (181 IBS patients, 133 HCs). Baseline characteristics are presented in Table 1. Fecal water ^1^H-NMR spectroscopic metabolic profiling data was obtained from 267 study individuals, and matching ^1^H-NMR and fecal MGS data were available from 262 study individuals (142 IBS patients, 120 HCs), for which the baseline data were not significantly different from that of the total study population. The MGS data has been published previously as a separate dataset in comparison with inflammatory bowel disease.^12^ In the present study, this MGS dataset has now been combined with novel measurement of fecal metabolites in the same fecal sample and studied in relation to extensive phenotype data of IBS patients. Multiple biomarkers were measured in subgroups of the total study population and have been published previously (now available for the current dataset in Table S1),^11,19–21^ showing in the current dataset significant differences in calprotectin, chromogranin A and human β-defensin 2 in fecal samples, plasma cytokines IL-1 and IL-10/12 ratio, and visceral sensitivity by rectal barostat between IBS and HC. No differences were found for plasma citrulline and the multi-sugar test for intestinal permeability. As for indicators of altered gut–brain interaction, in IBS patients higher scores for anxiety and depression, lower scores for the mental component of quality of life and high stress responsiveness (*i.e*. patients indicating that GI symptoms are triggered by stress in daily life and/or that IBS developed after a stressful life event) were found, when compared to HC (Table 1). Also altered levels of serotonin, 5-hydroxyindoleacetate (5-HIAA) and their ratio were measured in platelet-poor plasma of IBS patients, compared to HC (*Table S1, Figure S8*).

**Table 1.** **Baseline characteristics IBS patients versus HC** Differences tested with independent samples t-test and Pearson Chi^2^ as appropriate; *P < .05; **P < .01; ***P < .001 vs. HC. GI symptom diary, 14-day end-of-day diary on 1–5 Likert scale. GI Symptom Rating Scale (GSRS), end-of-week questionnaire on 1–7 Likert scale. HADS, 0–21 scale. SF-36 quality of life score, 0–100 scale. Diarrhea (IBS-D) and constipation (IBS-C) predominant subtype, mixed (IBS-M) and undefined (IBS-U) subtype. Hospital care patients are both from secondary and tertiary referral centers

Parameter	IBS (N = 181)	HC (N = 133)
Demographics and Lifestyle		
Age (mean years ± SD)	44.7 ± 16.9	45.8 ± 18.9
Female sex (%)	69.6*	58.6
BMI (mean kg/m^2^ ± SD)	25.0 ± 4.5	24.1 ± 3.9
Age at onset IBS (mean years ± SD)	31.6 ± 17.1	-
Duration of IBS symptoms (mean years ± SD)	12.5 ± 10.6	-
IBS subtype: IBS-D/IBS-C/IBS-M/IBS-U (n)	64/34/73/10	-
Acute onset of IBS, self-reported (%)	63.5	-
Post-infectious IBS, self-reported (%)	24.9	-
Current or previous smoker (%)	52.6***	39.7
Alcohol abstainers: 0 units/week (%)	40.7***	18.2
Moderate alcohol use: 1–15 units/week (%)	40.1**	47.0
Recruited via primary/hospital care (%)	17.7/82.3	-
**GI symptoms assessed by end-of-day diary, 14-day mean ± SD**		
Abdominal pain	2.3 ± 0.9***	1.1 ± 0.1
Abdominal discomfort	2.4 ± 0.8***	1.1 ± 0.2
Bloating	2.2 ± 1.0***	1.1 ± 0.2
Belching	1.7 ± 0.8***	1.1 ± 0.3
Nausea	1.6 ± 0.7***	1.0 ± 0.1
Flatulence	2.4 ± 0.9***	1.3 ± 0.5
Constipation	1.5 ± 0.7***	1.1 ± 0.2
Diarrhea	1.5 ± 0.6***	1.0 ± 0.1
Overall symptom burden	2.5 ± 0.8***	1.1 ± 0.2
**GSRS, mean ± SD**		
Abdominal pain syndrome	3.2 ± 1.2***	1.6 ± 0.7
Reflux syndrome	2.0 ± 1.4***	1.2 ± 0.5
Diarrhea syndrome	3.5 ± 1.5***	1.4 ± 0.7
Constipation syndrome	3.2 ± 1.4***	1.7 ± 0.9
Indigestion syndrome	4.0 ± 1.3***	2.0 ± 0.9
Total GSRS score	15.6 ± 4.1***	8.0 ± 2.6
**Psychological factors and Quality of Life**		
Onset of IBS after stressful event (%)	37.6	-
GI symptoms triggered by stress (%)	65.7	-
HADS anxiety score (mean ± SD)	6.9 ± 4.0***	3.7 ± 3.0
HADS depression score (mean ± SD)	4.4 ± 3.7***	2.2 ± 2.9
SF-36 physical QoL score (mean ± SD)	41.0 ± 10.3***	54.1 ± 5.7
SF-36 mental QoL score (mean ± SD)	47.4 ± 10.8***	53.7 ± 8.4

### Differences in fecal microbiome and metabolome data between HC and IBS patients

**Figure 1.**
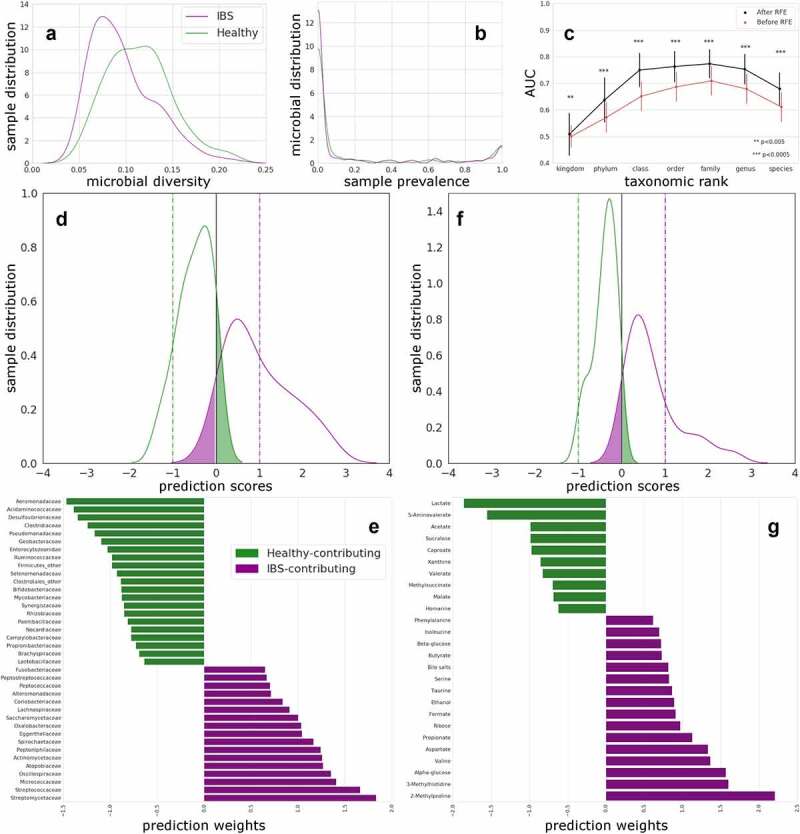
Distinguishing HC and IBS using microbiome at different taxonomic ranks, and using fecal water metabolites.

Gut microbial alpha diversity, measured by Simpson’s evenness metric, is significantly lower in IBS patients compared to HC (Figure 1a). As shown in Figure 1c, when considering different taxonomic ranks to differentiate between HC and IBS patients, not species-level, but rather family-level differentiated best between the two groups. The differentiation between HC and IBS patients could be improved further by use of recursive feature elimination (RFE) to an area-under-the-receiver-operator-curve (AUC) of 77.3% (Figure 1d) for the family-level taxonomic rank. The bacterial families responsible for this differentiation are shown in Figure 1e.

**(A)** Diversity distribution, measured by the Simpson’s evenness metric, of the IBS (purple) and HC (green) cohorts is significantly (*P* = 1.01 × 10^−6^) different according to a Mann Whitney test. **(B)** Microbial prevalence distribution in HC and IBS; no significant difference is found. Graphs shows distribution of relative abundance of microbes (in %) across the HC and IBS samples. 0 indicates 0% and 1 indicates 100% of the individual microbes present. **(C)** AUC based on the different taxonomic ranks (indicated on the x-axis) for IBS versus HC, with standard deviation intervals, shown for the linear SVC models with (black) and without RFE (red). Significance is indicated for each paired (before vs after RFE) test. **(D)** Predicted scores as kernel density estimates from SVC models comparing IBS with HC (family-level data after RFE). Testing data separation from decision boundary. False positives are presented in the green shaded area of the curves and false negatives on the purple. Horizontal lines are drawn to indicate the optimal hyperplane (x = 0, black) and maximum margin (x = −1, HC; x = 1, IBS). **(E)** Bacterial relative abundance at family level presented as SVC weights for HCs and IBS patients. Twenty-one families, presented as SVC weights, were more abundant in HCs (in green) and 17 in IBS patients (in purple). **(F)** Predicted scores as kernel density estimates from SVC models comparing IBS with HCs for fecal metabolite data after RFE. Testing data separation from decision boundary. False positives are presented in the green shaded area of the curves and false negatives on the purple. Horizontal lines are drawn to indicate the optimal hyperplane (x = 0, black) and maximum margin (x = −1, HC (green); x = 1, IBS (purple)). **(G)** Fecal metabolites increased in HCs (green, negative weights) and in IBS patients (purple, positive weights) and contributing to the differentiation between the two groups in the SVC model.

From the ^1^H-NMR spectrum, 50 unique metabolites have been identified in fecal water of IBS patients and HC (Figure S1). Based on these metabolites, HC and IBS patients could be differentiated with an AUC of 79.5% (figure 1f). The metabolites responsible for this differentiation are shown in Figure 1g; 10 metabolites were increased in HCs and 16 were higher in IBS patients.

### Multi-omics integration to predict differences between HC and IBS

**Figure 2.**
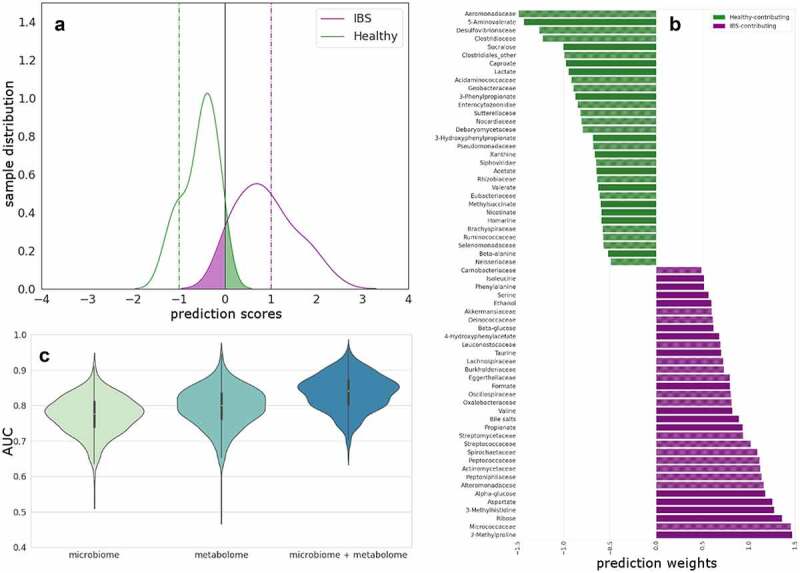
Multi-omics integration to predict differences between HC and IBS.

When combining both family-level fecal microbiota and fecal metabolites the two groups can be differentiated with an AUC of 83.6% (Figure 2a). This model consists of both microbial families and metabolites that differentiate between HC and IBS patients (Figure 2b). As shown in Figure 2c, compared to using only the microbiota or fecal metabolites to differentiate between groups, the combination of both omics levels provides the best differentiation between HC and IBS patients.

**(A)** Combined linear RFE for IBS versus HC. Predicted scores shown as kernel density estimates from SVC models comparing IBS with HCs after RFE. Testing data separation from decision boundary. False positives are presented in the green shaded area of the curves and false negatives on the purple. Horizontal lines are drawn to indicate the optimal hyperplane (x = 0, black) and maximum margin (x = −1, HC (green); x = 1, IBS (purple)). **(B)** Combined fecal metabolites and microbiota families increased in HCs (green) and in IBS patients (purple) and contributing to the differentiation between the two groups in the SVC model. **(C)** Beanplots showing statistically significant differences in the AUC between models distinguishing IBS patients from HC based on microbiota-family, fecal metabolites and the combination of both. The corrected *P*-value for metabolome versus combined is 2.90 × 10^−100^ and microbiome versus combined is 2.77 × 10^−56^ (Wilcoxon test and FWER multiple correction).

Furthermore, when considering the combined data, pairwise Mann–Whitney U-tests did not show statistically significant differences in the predicted scores (Figure S2) between the three classical IBS subtypes based on Rome III criteria (constipation, diarrhea predominant and mixed subtype).

### Clustering of IBS patients based on fecal microbiota and metabolites and the relation to clinical and biological features

**Figure 3b.**
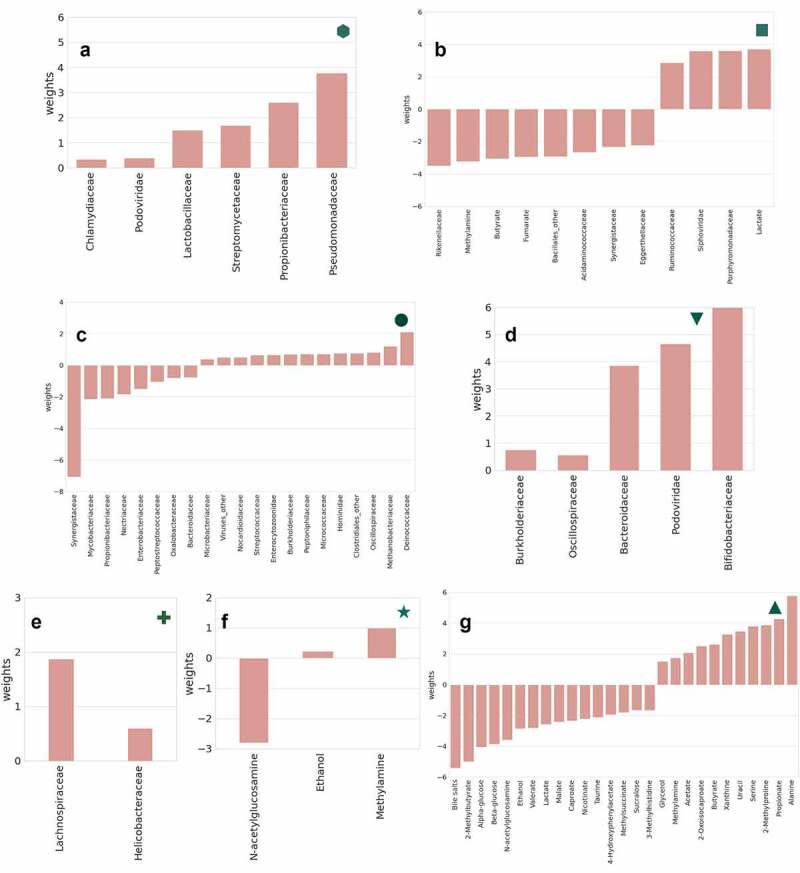
Important features for SVR/SVC models of clinical markers significantly different between clusters.

When considering only IBS patients, different clusters of patients can be identified based on fecal microbiota and metabolites separately and by combining both omics data (Figures 3a , b, S3). These clusters were then investigated with respect to differences in extensive clinical and biological metadata available in this cohort (Figure 3a); after multiple testings, these include duration of IBS symptoms, GI symptoms triggered by stress, onset of IBS after a stressful event, plasma 5-hydroxytryptamine (serotonin) and 5-hydroxyindoleacetate, total dietary energy intake and medical history of abdominal surgery (including appendectomy or cholecystectomy). For each of these pathophysiological and clinical features of IBS patients, the important features (microbial families and/or metabolites) from subsequent support vector machine classifier (SVC)/support vector regressor (SVR) models are given in Figure 3a, b.
**Figure 3a.**
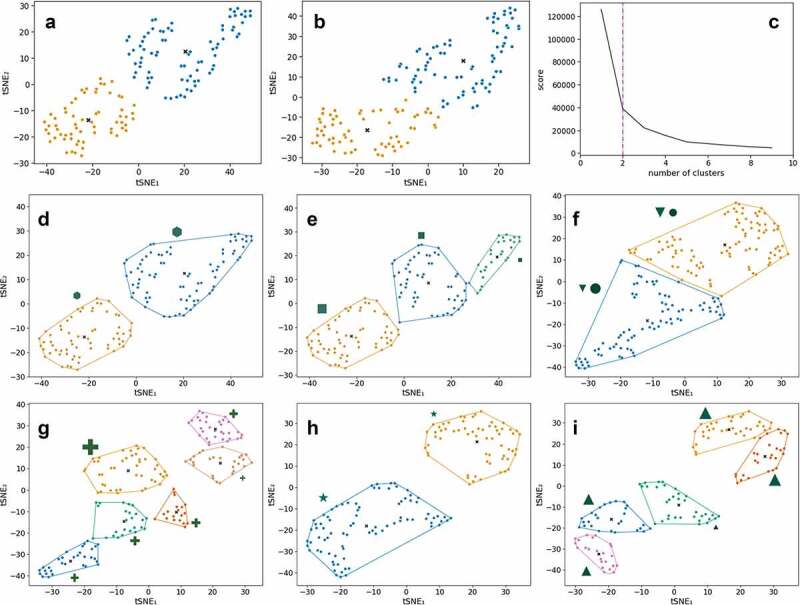
Unsupervised clustering using kernel t-SNE (kt-SNE)

**(A-B)** Two selected kt-SNE mappings from 1,000 individual t-SNE models with the optimal clusters indicated in colors (combined data of fecal metabolites and microbiota families). **(C)** Elbow plot of the average scores across the 1,000 individual kt-SNE models (combined data of fecal metabolites and microbiota families). **(D-I)** Subgroup analyses of clusters from kt-SNE analyses for which clinical/meta data variables were most significantly different between groups (see Figure S5 for results for all three datasets and all number of clusters, corrected for multiple testing). The relative size of the markers indicates the average value of the variable in the cluster. Each cluster is surrounded by the convex hull in the same color. **(D)** Duration of IBS symptoms (**hexagon**, *P*_adj_= 1.87 × 10^−2^) is significantly different between the two clusters in the combined data of fecal metabolites and microbiota families. **(E)** IBS symptoms triggered by stress (**square**, *P*_adj_= 3.67 × 10^−3^) is significantly different between the three clusters in the combined data of fecal metabolites and microbiota families. **(F)** Onset of IBS after stressful event (**circle**, *P*_adj_= 8.17 × 10^−12^) and serum 5-hydroxytryptamine (serotonin) (**down-pointing triangle**, *P*_adj_= 2.79 × 10^−2^) are significantly different between two clusters in the microbiome family-level data. **(G)** Sum of total dietary energy based on the food frequency questionnaire (**cross**, *P*_adj_= 4.15 × 10^−2^) is significantly different between six clusters in the microbiome family-level data. **(H)** 5-hydroxyindoleacetic acid in poor platelet plasma (**star**, *P*_adj_ = 3.22 × 10^−2^) is significantly different between two clusters in the fecal metabolite data. **(I)** Medical history of abdominal surgery (**upward-facing triangle**, *P*_adj_= 3.86 × 10^−2^) is significantly different between five clusters in the fecal metabolite data.

**(A, hexagon)** Microbiota families associated with the duration of IBS symptoms in this cohort (no metabolites are associated but were included in model). **(B, square)** Fecal metabolites and microbiota families associated with IBS symptoms triggered by stress. **(C, circle)** Microbiota families associated with onset of IBS after stressful event. **(D, down-pointing triangle)** Microbiota families associated with 5-hydroxytryptamine (serotonin) in poor platelet plasma. **(E, cross)** Microbiota families are associated with the sum of total dietary energy (from food frequency questionnaires). **(F, star)** Fecal metabolites associated with 5-hydroxyindoleacetate in poor platelet plasma (marker of serotonin metabolism). **(G, upward-facing triangle)** Fecal metabolites associated with medical history of abdominal surgery in this IBS patient cohort.

In this cohort, we previously, in larger datasets, measured visceral hypersensitivity (using rectal barostat), intestinal permeability (using a multi-sugar test) and intestinal inflammation (by fecal calprotectin).^11,20,23^ However, these mechanisms were not identified as relevant in the unsupervised analyses here. This may be related to missing data, as we conducted these measurements only in subgroups of IBS patients in the present study. Exploratively, in a posthoc analysis (Figure S4), we identified specific gut microbial families and metabolites associated with these presumed pathophysiological mechanisms. Several bacterial families, which may be considered proinflammatory in the gut, were increased in subjects with higher fecal calprotectin levels. With regard to intestinal barrier measurements, several bacterial families related to proteolytic fermentation and microbes previously associated with intestinal barrier modulation,^24^ such as *Bifidobacteriaceae*, were associated with increased intestinal permeability. No significant associations with microbiota or metabolites were found in relation to visceral hypersensitivity.

### Metabolic reaction network

**Figure 4.**
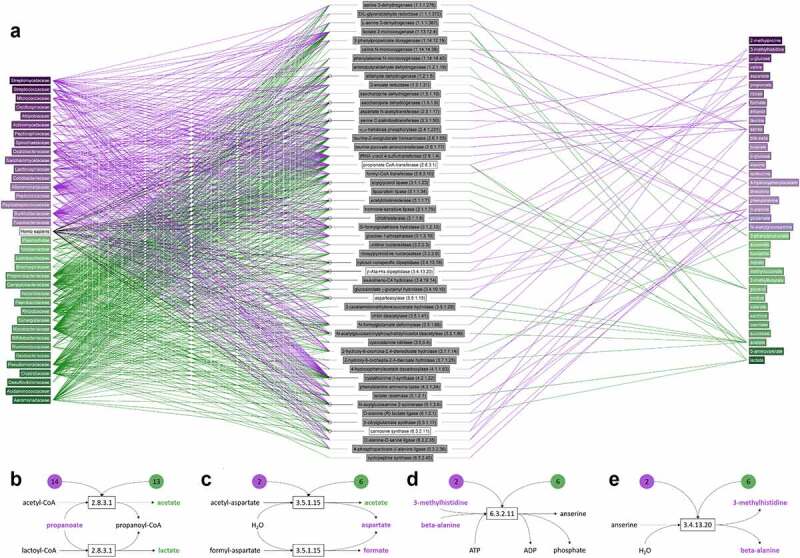
**Metabolites involved in reactions mediated by enzymes found in one or more of the microbial families**. (a) Enzymatic reactions from all microbial species in the KEGG database that belong to the families that were identified in our microbiome analysis were included in this figure. Two metabolites were considered associated with each other if a biochemical reaction entry in KEGG indicates that they are a main reactant pair and the enzyme involved is linked to a human or microbial gene. Left column shows microbial families and *Homo sapiens* for reference, the right column the metabolites and the middle column the enzymes (enzyme code) that are encoded by genes from microbiota (and *Homo sapiens*) that mediate reactions involving one or more metabolites on the right. Microbial families and metabolites in green are increased in HCs and purple in IBS patients. This representation is based on the full metabolic reaction network in Figure S5. (b-e) Four reactions mediated by microbial enzymes that involve two or more metabolites associated with HC (green) or IBS (purple). The numbers in green and purple indicate the number of microbial families that have a gene encoding this enzyme. For web-based access of the Figure 4a, please check on the link https://imperialcollegelondon.box.com/s/ka1f3c3g64t0t1p0p6g4hgys0friaghm. In which you can point at any dot in the graph and all the lines attached to that dot are made visible. This provides perfect insight into our data and any connections that are present between different datasets.

In order to identify links between gut microbiota and corresponding metabolites, an extensive metabolic reaction network was constructed involving significantly increased and decreased gut microbial families and fecal metabolites in IBS patients (full network in Figure S5). A representation based on this network is presented in Figure 4a. In this interactive figure, the microbial families found to be increased in IBS (purple) and increased in HC (green) are shown on the left. In the figure, via the centralized enzymes, the pathways toward the fecal water metabolites on the right, again in purple increased in IBS, and green increased in HC, can be followed. Pathways for saccharolytic and proteolytic metabolic activity can be extrapolated from this graph. First, propionate-CoA transferase, a microbial enzyme involved in fatty acid synthesis and oxidation, found in 14 IBS- and 13 HC-associated bacterial families, can produce both acetate and lactate (both higher in HC, Figure 4b). CoA-bound forms of acetate or lactate are released and CoA-bound propionate is formed from free propionate (higher in IBS). Second, a general class of aspartoacylases that produce aspartate plus carboxylate from an acylaspartate-substrate (Figure 4c). The carboxylic acid metabolites, formate, and acetate can be produced by reactions mediated by this enzyme. Fecal aspartate and formate are both higher in IBS, whereas acetate is higher in HC. Specifically, *N*-formylaspartate amidohydrolase (*Homo sapiens* and microbial enzyme) produces both aspartate and formate. Six HC-associated microbial families (*Aeromonadaceae, Clostridiaceae, Mycobacteriaceae, Rhizobiaceae, Campylobacteraceae, Propionibacteriaceae*) have this enzyme and also 2 IBS-associated families (*Alteromonadaceae, Burkholderiaceae*). Third, alanine-lactate ligase, found in 5 HC and 3 IBS associated microbiota families, uses both alanine and lactate as substrates. However, alanine is found in higher concentrations in IBS and lactate higher in HC. A number of enzymes catalyze reactions involving multiple metabolites that are all associated with either IBS or HC. Lactate 2-monooxygenase produces acetate from substrate lactate, both higher in HC. However, valine *N*-monooxygenase can use both valine and isoleucine as substrate, both these branched-chain amino acids (BCAAs) are higher in IBS and can only be found in some species within the *Mycobacteriaceae* family. Last, carnosine synthase produces anserine from substrates beta-alanine and 3-methylhistidine (both higher in IBS, Figure 4d), whereas beta-alanine-histidine dipeptidase catalyzes the reverse reaction (Figure 4e). *Homo sapiens*, HC-associated *Aeromonadaceae, Clostridiaceae, Mycobacteriaceae, Rhizobiaceae, Campylobacteraceae* and *Propionibacteriaceae*, and IBS-associated *Alteromonadaceae* and *Burkholderiaceae* microbial families all have both of these enzymes.

## Discussion

This study was performed to provide new insights into complex microbiota–host interactions in IBS, using a systems biology approach. We incorporated in-depth analyses of multiple omics layers in a cohort of well-phenotyped clinically diagnosed IBS patients and HCs, taking into account pathophysiological and clinical features related to inflammation and neurohormonal activity.

While both the fecal microbiome, which was shown previously,^25^ and fecal metabolome, which was now measured for the first time, could be used to distinguish IBS and healthy individuals, the prediction could substantially be improved by combining these data in the current study. Interestingly, we now also showed that classical, stool pattern-based, IBS subtypes could not be discriminated from each other based on the microbiome–metabolome data. However, by use of unsupervised clustering analyses, distinct subsets of IBS patients based on fecal microbiome–metabolome data were identified. These newly identified clusters of IBS patients based on gut microbiome–metabolome data and potential pathophysiological mechanisms could not only guide further research but also provide clues for new options for treatment in the future. The gut microbiome–metabolome clusters identified in the present study were associated with the following potential, previously identified, pathophysiological mechanisms in IBS: effects of psychological stress on GI symptoms and onset of IBS after stressful events, plasma levels of serotonin and its metabolite 5-hydroxyindoleacetate, medical history of previous abdominal surgery, dietary caloric intake and time since IBS diagnosis. Furthermore, a complex metabolic reaction network was integrated with microbiome information to better understand microbiota and metabolite interactions in IBS. These findings provide additional insight into potential mechanisms underlying the host–microbiome interaction in IBS.

A lower microbial alpha biodiversity is considered less beneficial for human health^26^ and has previously been shown in IBS;^12,27,28^ here, we again find it was decreased in IBS patients. However, other than microbiota composition, different gut microbes also frequently share similar metabolic capacities. Despite temporal differences in microbiota composition, the microbiome’s metabolic function has been shown to be quite stable over time,^29^ indicating that microbial metabolic function may be superior to microbial composition when studying the relationship between the microbiome and pathophysiology of disorders.^26^ When considering the most pronounced differences in the combined microbiome–metabolome analysis between IBS and HC in the current study, we found relevant indications for altered microbiota–host interactions. There was an increased relative abundance of *Streptomycetaceae* in IBS, a family in the phylum of *Actinobacteria*. The members in this family are known for their ability to produce important secondary metabolites in the arginine and BCAA-pathways.^30^ BCAA valine and isoleucine, both increased in fecal water of IBS, can serve as a substrate for valine *N*-monooxygenase, found in some species within the *Mycobacteriaceae* family, which were also increased in IBS. Previously, both these bacterial families were shown to be increased in oral mucosa of IBS patients and were associated with visceral sensitivity.^31^ Proton pump inhibitors (PPI), frequently used by IBS patients, can alter the fecal microbiome by increasing abundances of oropharyngeal microbes.^32^ In the present study, medication use, including PPI, was taken into account, and none of the identified clusters of IBS patients based on the microbiome–metabolome data were significantly associated with PPI use.

We also observed an increased level of 2-methylproline, one of the precursors for de novo synthesis of arginine in the intestine and involved in glutamate metabolism.^33^ These pathways are components of the microbial proteolytic activity in the colon and may point toward alterations in this network in IBS patients. This was also found for other amino acids, such as phenylalanine and serine involved in proteolytic fermentation, and bacteria which can produce these amino acids, such as *Peptococcaceae*. Proteolytic metabolic activity is most pronounced in the distal part of the colon and is considered detrimental for colonic and metabolic health.^34^ While saccharolytic metabolic activity, occurring predominantly in the proximal colon, is considered beneficial for human health, it yields high amounts of short-chain fatty acids (SCFAs), as well as lactate and succinate. While in the present study, the SCFA propionate was increased in IBS, other SCFAs such as caproate, acetate and valerate were decreased. The same was true for lactate, which is a product of fermentation of dietary fiber.^34^ Furthermore, *Clostridiaceae* and *Ruminobacteriacceae*, both SCFA-producing bacteria, were also decreased in IBS patients. Although in this cross-sectional design causal relationships cannot be investigated, these microbial abundances and metabolites point toward decreased saccharolytic and increased proteolytic metabolic activity in IBS patients. Increased fecal proteolytic activity has also previously been shown in IBS patients, especially in the post-infectious subgroup.^35^ It has to be noted that in the current study, post-infectious IBS could not clearly be identified based on the available data.

Diet plays an important role in the gut microbial metabolic activity. We used data from an extensive food frequency questionnaire, as published previously.^22^ When these data were combined in the current dataset, we found that total caloric intake was associated with higher relative abundances of *Helicobacteraceae*, previously associated with colonic inflammation,^36^ and *Lachnospiraceae*, one of the main producers of SCFAs in the colon.^37^ Although extensive data on dietary intake was available, we did not find further associations with other data. This may in part be explained by use of a questionnaire to assess dietary patterns over the last month rather than the day prior to sampling.

Altered gut–brain interaction is considered the key pathophysiological mechanism in IBS.^8^ We have previously shown that IBS patients do not experience higher daily life stress than controls but do have an exaggerated stress response to GI symptoms.^38^ Also, in our current data, 66% of IBS patients noted a strong relation between daily psychological stress and GI symptoms. Furthermore, 38% indicated that their IBS developed after a stressful life event. Furthermore, altered gut microbiota-mediated activation of several neurotransmitter systems, including serotonin metabolism and the hypothalamic-pituitary-adrenal axis, are assumed to play an important role in the dysregulated gut-brain interaction in IBS.^9^ Previously, we showed that serotonin metabolism is altered in patients with IBS and is associated with alterations in bowel movements.^21^ To add further evidence on the microbiota-gut-brain link in IBS, in the present study, we identified clusters of IBS patients based on microbiome–metabolome profiles, which were associated with altered systemic levels of serotonin, its metabolite 5-hydroxyindoleactic acid, as well as the presence of stress-related GI symptoms. In our previous study, in all IBS subtypes, plasma 5-HIAA was lower compared to HCs, but this was most pronounced in the IBS-M subtype, in which also the 5-HIAA/5-HT ratio was decreased.^21^ In the present study, the identified clusters based on fecal microbiota–metabolome data associated with the plasma levels of serotonin and 5-HIAA in IBS could not be linked to a specific IBS subtype. Furthermore, serotonin and its metabolites were not identified in fecal water in the present study. Although the mechanisms underlying altered plasma serotonin levels in IBS are incompletely understood, it is known that serotonin and its metabolites not only modulate the central nervous system and thereby psychological and behavioral processes, but have also key modulatory functions in the enteric nervous system, co-regulating intestinal secretion, motility and visceral perception.^39^ In the present study, *Bifidobacteriaceae, Bacteroidaceae* and *Oscillospiraceae*, but also the bacteriophages *Podoviridae*, of which *Bacteroidaceae* was the natural host, were associated with increased plasma serotonin levels. Interestingly, these bacterial families were previously associated with depression, a psychiatric disorder in which altered serotonin metabolism plays a key role.^40^ Previous associations with depression were also shown for *Acidaminococcaceae, Eggerthellaceae* and *Rikenellacea*e,^40^ which we found decreased in IBS patients with pronounced stress-GI symptom interactions in the present study. Increased abundance of *Bifidobacterium* species was previously associated with microbiota dysbiosis in mice with serotonin transporter deficiency.^41^

We also found *Porphyromonadaceae* and *Ruminococcaceae* abundances increased in IBS patients with altered stress sensitivity. These bacterial families were previously associated with psychological stress accompanied gut microbial dysbiosis in mice,^42^ possibly secondary to altered immune activity. In addition, *Streptococcaceae, Microccoceae* and *Actinomycetaceae* families were increased in IBS patients in our study, which is in line with previously found higher abundances of these families in patients with abdominal pain.^43^ Furthermore, *Streptococcaceae* have been shown in culture to synthesize serotonin, and animal studies have demonstrated a strong regulatory effect of gut microbiota and both systemic and intestinal serotonin host pathways.^44^

Interestingly, while our findings point toward an altered interaction between the gut microbiome and systemic neurohormonal activity and stress reactivity in IBS patients, we found no associations with anxiety and depression (*i.e*. Hospital Anxiety and Depression Scale (HADS) scores). It should be noted that HADS is merely a screening stool both for anxiety and depression, and although it correlates well with other questionnaires for these psychological conditions in IBS,^45^ it has specific limitation and does not cover the full spectrum of symptoms. Further mechanistic studies are needed to identify any potential causal relationships between gut microbial metabolic activity, altered serotonin metabolism and stress responsiveness; our current findings provide new leads for improved understanding of key pathways along the microbiome-gut-brain axis in IBS.

Previous studies showed that bile acid malabsorption is present in almost 30% of IBS patients, in particular those with the diarrhea predominant subtype.^46^ Metabolites taurine and glycine interact with free bile acids to compose bile salts. We found both taurine and bile salts were increased in fecal samples of IBS patients. Bile salts were particularly increased in the diarrhea predominant subtype, but after correction for multiple testing, this association was no longer significant.

Furthermore, gut microbial and metabolic profiles in IBS patients were associated with a medical history of abdominal surgery, including uncomplicated appendectomy or cholecystectomy. Earlier studies pointed to alterations in gut microbiota composition after these surgical operations.^47,48^ Furthermore, appendectomy has been shown to increase the risk of developing IBS, possibly as a consequence of gut microbiome changes.^49^

Studying the gut microbiome and functional relevance of observed perturbations in composition remains challenging. Fecal sampling is noninvasive, readily accessible and therefore feasible to study the gut microbiome in large study populations. It is by far the most used biomaterial for microbiota research, yielding high potential of comparability. Furthermore, fecal microbiota composition is assumed to provide a representative sample of gut lumen spatial variation. However, differences between gut microbiota composition present in the gut lumen, mucus samples and mucosal biopsies have been described.^50^ Whether this is a consequence of sampling methodology or biologically relevant differences between sampling sites remains a matter of debate. Nevertheless, it may explain differences observed between studies. Also identifying metabolites in fecal water is challenging. We used standardized procedures to homogenize as good as possible a by nature non-homogenous fecal sample and to prepare the sample for analysis. For the NMR fecal water data, we aimed to identify as many metabolites as possible using both statistical analyses, in-house databases and spike-in experiments and then integrated all peaks (including unknown/unidentified signals). We used only those signals from metabolites that were identified with confidence for the main analysis.

Furthermore, it has to be noted that due to the case-control design of the current study, causal effects could not be delineated. Also, the final number of included subjects in the present study was based on available overlapping datasets to perform the extensive systems biology approach, which limited any sample size calculation. Our results merit replication in independent cohorts and longitudinal analysis would be of additional benefit. However, the major strength of our work is the simultaneous assessment of fecal microbiota composition, its metabolic (genetic) potential and metabolic activity in a deeply phenotyped well-characterized group of IBS patients, and these unique observations revealed numerous starting points for new mechanistic research.

## Conclusion

Fecal microbiome–metabolome signatures have been identified for IBS, which were associated with altered serotonin metabolism and unfavorable stress response related to GI symptoms. Metabolic reaction networks integrated with microbiome information show pathways for the host–microbiome interaction. These results support the microbiota-gut-brain link in the pathogenesis of IBS.

## Methods

### Study participants; MIBS cohort

Participants of the MIBS cohort^11^ were included in this case-control study. The research protocol has been approved by the Maastricht University Medical Center+ (MUMC+) Ethics Committee and is registered in the US National Library of Medicine (NCT00775060). Patients or the public were not involved in the design, conduct, reporting or dissemination plans of our research. The STORMS checklist has been completed (Supplementary Materials).^51^

All participants gave written informed consent prior to participation. IBS patients, between 18 and 75 years of age, were recruited via the outpatient gastroenterology–hepatology clinic of MUMC+, a secondary and tertiary referral center in Maastricht, the Netherlands, and via general practitioners in the area of this hospital, all diagnosed according to Rome III criteria. The HC group were enrolled via public advertising. Study participants whose fecal samples and phenotype data that were available were included in the present study. Detailed descriptions of the inclusion process, collected questionnaires on GI symptoms, psychological factors, diet,^22^ medication use, quality of life, and measurements on intestinal barrier function,^20^ visceral hypersensitivity,^19^ and biomarkers in faces and blood plasma related to inflammation and neuroendocrine activity,^11,21^ have been published elsewhere and a short description is included in the Supplementary Materials. All participants were asked to produce a fecal sample at home, place it in their refrigerator (4°C) and bring it to the hospital on cold packs within 24 hours. Here, the samples were collected and aliquots were made and stored at −80°C until analysis. The use of antibiotics was noted, and fecal samples that were not used for microbiota analysis or fecal metabolic profiling in case antibiotics were used in 3 months prior to sample collection. Any shipment of samples was carried out on dry ice and did not last longer than 48 hours.

### Microbiota analysis using whole-genome shotgun MGS

A detailed description of the DNA extraction and MGS has been published previously.^12^ In short, Fecal DNA isolation was performed using the AllPrep DNA/RNA Mini-Kit (Qiagen;cat.#80204) with the addition of mechanical lysis as described previously.^52^ After fecal sample collection and DNA extraction, fecal DNA was sent to the Broad Institute of Harvard and MIT in Cambridge, Massachusetts, USA, where library preparation and MGS were performed on the Illumina HiSeq platform. From the raw MGS data, low-quality reads were discarded by the sequencing facility using an in-house pipeline. Samples with a read depth under 10 million reads were excluded from subsequent analyses. Next, quality trimming and adapter removal were performed using Trimmomatic^53^ (v.0.32). The MGS data was processed and taxonomic labels were assigned using Kraken.^54^ Bacterial, viral and eukaryote abundances were determined for patients and controls. This was performed using fecal samples of 178 IBS patients and 133 HCs. Three samples were excluded due to low data quality or unsuccessful sequencing.

### Fecal metabolic profiling

Fecal water was obtained using 1.0 gram of homogenized fecal sample in accordance with a previously published standardized protocol.^55^ It was analyzed using ^1^H-NMR spectroscopy in line with the same protocol.^55^ All NMR experiments were acquired at 300 K using a spectrometer operating at 600.29 MHz for ^1^H and equipped with a 5 mm BBI Z-gradient probe (Bruker BioSpin, Karlsruhe, Germany) with a standard one‐dimensional pulse sequence (noesygppr1d). Individual peaks were integrated and identified *a priori* using statistical methods,^56^ in-house databases and additional experiments.^57^ Resolved peaks that could not be identified were included in separate analyses, with identified metabolites included in the main analyses. The total number of identified metabolites in these data was 49, with a further 61 signals remaining unidentified. The individual spectra were quality checked by a spectroscopist. To ensure that each spectrum was acquired in a high-quality manner, we followed a systematic quality assurance criterion as follows: a line width at half height of the internal standard (TSP) < 1.5 Hz (line command “peakw” or hwcal macro), flat baseline within the range of all the spectral width used, consistency in the quality of the water suppression, no phase errors and an adjusted and same receiver gain. When a spectrum does not meet this criterion, the sample was re-run. More details on sample preparation, analysis and data processing can be found in the Supplementary Materials. ^1^H-NMR outliers were excluded based on Hotelling’s T^2^ statistic of unsupervised dimension reduction (Figure S6), and spectra were processed to remove baseline artifacts (Figure S7). All but two of the outliers identified contained high concentrations of polyethyleneglycol, and these were removed from any further analysis. The global profile of the remaining spectra (n = 267) was not significantly different between these and with comparable dilution of metabolites. The untargeted NMR methodology used (with a standard NOESY pulse sequence) does not allow going beyond semi-quantitative/relative measures of these metabolites, which allows to compare them in a multivariate model (to compare the data with itself) but does not allow to report concentrations (mmol/ml or mmol/mg dried weight).

### Data, statistical and bioinformatic analysis

All analyses were performed on microbiome and metabolome data separately, and on both datasets combined. SVCs^58^ were utilized to develop models for classifying IBS and HC binary classes using a linear kernel. The classifiers were trained 1,000 distinct times, where each time random training and test splits (80:20 ratio) were done *ab initio*. Splits of sets were tracked to ensure that the same train-test split was assigned across different models so as to allow for robust modeling. All input features were auto-scaled (mean-centering of each feature followed by division by standard deviation). A grid search with 5-fold cross validation was used to find optimal cost hyperparameter values that maximized accuracy for each model; 13 values were evaluated, starting from 0.001 and increasing on a log-scale. Hyperparameter tuning for the models was performed twice, before and after feature selection. Feature selection for SVC models was performed using RFE^59^ of feature weights. The sign of a feature weight indicates the association with either IBS or HC. Each model’s accuracy and effectiveness were evaluated using AUC score.

A combination of unsupervised learning methods was used to explore potential subgroupings within the IBS patients. A three-step pipeline was developed by using dimensionality reduction via kernel t-distributed Stochastic Neighbor Embedding (kt-SNE),^60^ k-means clustering and univariate testing to assess the resulting groups. kt-SNE was used to reduce the dimension of data from over 200 microbiome and metabolomic variables to a 2-dimensional coordinate system as input to k-means clustering.

The within-cluster sum-of-squared errors (WSS) were used to find the optimal number (2 ≤ k ≤ 10) of clusters for the k-means algorithm. The optimal value was determined using the elbow method for the highest second-order difference in WSS. Across 1,000 kt-SNE models and for k = 2–10, cluster membership of each sample was determined by calculating a proximity matrix indicating the frequency of sample-sample pairs ending up in the same cluster (for k clusters). The proximity matrix (one for each different k) was then analyzed using hierarchical clustering to find k groups of minimum size 5. These robust groups, combined over 1,000 kt-SNE models, were used as input for the univariate testing.

At the third step, a number of non-parametric statistical tests were employed to assess statistical significance of the resulting groups. The Kruskal–Wallis test was used to determine statistically significant differences between clusters (when k > 2), followed by a Mann–Whitney U-test to assess all sets of cluster pairs. These tests were applied on over 200 phenotypic variables including demographic characteristics, lifestyle and dietary features, GI symptoms and Rome III subtypes. These results were adjusted for multiple testing using the Hommel correction across all clinical data.

SVCs and SVRs were used to find microbiome and metabolic features that associate with phenotypic variables that were statistically significantly different between groups. SVC/SVRs were run for binary classification/continuous regression problems on clinical data variables that showed significance in the unsupervised kt-SNE clustering models. All analyses were performed in Python (v3.7) using scikit-learn (v0.23.2), pandas (v1.1.3), matplotlib (v3.3.2) and NumPy (v1.19.2) libraries.

Metabolic reaction networks were constructed based on the MetaboNetworks software^61^ (v2.3) and the KEGG database of biochemical reactions that occur in the human supra-organism. In addition to reactions mediated by human enzyme-coding genes, here we included enzymatic reactions from all microbial species in KEGG that belong to the families that were identified from the microbiome analyses. The constructed database considers that two metabolites are associated with each other if a biochemical reaction entry in KEGG indicates that they are the main reactant pair and the enzyme involved is linked to a human or microbial gene.

More details of all methods can be found in the Supplementary Materials.

## Supplementary Material

Supplemental Material

## Data Availability

The raw sequence MGS data sets, and age and gender information per sample are available on the European genome-phenome archive (https://www.ebi.ac.uk/ega/) at accession number EGAD00001002668. The fecal metabolite data has been made available on Figshare.com via the link: https://doi.org/10.6084/m9.figshare.19251599.v1
