# Supplementary material for: Integrated fecal microbiome–metabolome signatures reflect stress and serotonin metabolism in irritable bowel syndrome
Source: Gut Microbes. 2022 Apr 21;14(1):2063016. doi: 10.1080/19490976.2022.2063016 (PMC9037519; doi:10.1080/19490976.2022.2063016)
Supplement: Supplemental Material [file KGMI_A_2063016_SM2904.zip › Supplementary Materials.docx]

**Supplementary Materials**

**Integrated Faecal Microbiome-Metabolome Signatures Reflect Stress and Serotonin Metabolism in Irritable Bowel Syndrome**

**Zlatan Mujagic^1,2,3*^, Melpomeni Kasapi^3*^**, Daisy MAE Jonkers^1,2^, Isabel Garcia-Perez^4^, Lisa Vork^1,2^, Zsa Zsa R.M. Weerts^1,2^, Jose-Ivan Serrano-Contreras^4^, Alexandra Zhernakova^5^, Alexander Kurilshikov^5^, Jamie Scotcher^3^, Elaine Holmes^4,6^, Cisca Wijmenga^5^, Daniel Keszthelyi^1,2^, Jeremy K Nicholson^6^, **Joram M Posma^3#^, Ad AM Masclee^1,2#^**

* / ^#^ authors share co-first and co-last authorship, respectively

***Extended methods:* Study population**

All IBS patients were diagnosed by their gastroenterologist or general practitioner by use of Rome III criteria. When considered indicated by the treating physician GI endoscopy with biopsies, abdominal imaging and/or blood, breath and faecal analyses were performed for exclusion of organic disease. All patients with a history of major abdominal surgery, apart from uncomplicated appendectomy, laparoscopic cholecystectomy and hysterectomy, were excluded. IBS subtypes were based on predominant bowel habits according to the Rome III criteria: diarrhoea (IBS-D), constipation (IBS-C), mixed stool pattern (IBS-M) and unspecified subtype (IBS-U). Demographic characteristics and lifestyle factors were reported by all study participants using standardized questionnaires. Use of medication and medical history were self-reported and were crosschecked using hospital medical records. GI symptom were scored on a 14-day end-of-day diary, addressing abdominal discomfort and pain, nausea, bloating, belching, flatulence, diarrhoea and constipation, on a 1-to-5 point Likert scale. During the same 14-day period stool frequency and consistency was assessed daily using the Bristol Stool Chart. Furthermore, at the end of that period, GI symptoms were measured, on a 1-to-7 point Likert scale, with a one-week recall period, by the Gastrointestinal Symtom Rating Scale (GSRS). Diet was assessed using a validated food frequency questionnaire.([Tigchelaar et al., 2017](#_ENREF_49))

Intestinal permeability was measured using a validated multi-sugar test.([Mujagic et al., 2014](#_ENREF_35)) Visceral hypersensitivity was assessed using rectal barostat.([Ludidi et al., 2014](#_ENREF_31))

***Extended methods: measurement of biomarkers in blood and faecal samples***

Blood samples were collected using standardized collection procedures in K_2_EDTA (BD Vacutainer®) tubes and centrifuged to obtained plasma supernatants, which were aliquoted and frozen at -80 °C until analysis.

At the same time period, subjects collected stool samples, stored it at home at 4 °C and brought to the hospital within 24 hours. The samples were then aliquoted and stored at -80 °C within 24 hours after collection. The use of antibiotics was noted, and faecal samples where not used for microbiota analysis in case antibiotics where used in 3 months prior to sample collection. Samples were shipped on dry ice to the laboratory for analysis, and the shipment did not last longer then 48 hours.

Several biomarkers were measured in blood and faeces as described previously.([Mujagic et al., 2016](#_ENREF_36); [Thijssen et al., 2016](#_ENREF_48)) In faecal samples calprotectin, an indicator of intestinal inflammation that is proportional to neutrophil migration towards the intestinal tract, human beta defensin-2 (HBD2), a human antimicrobial peptide produced by among others intestinal epithelial cells in response to (pathogenic) bacteria, chromiogranin-A (CgA), an indicator of the intestinal neuroendocrine cell activity that is co-localized in storage granules in enterochromaffin cells with serotonin, and short chain fatty acids, *i.e.* acetate, propionate, butyrate, valerate and caproate, were measured using enzyme-linked immunosorbent assay (ELISA), radioimmunoassay (RIA), and gas chromatography-mass spectrometry (GC-MS), respectively. In blood plasma cytokines, *i.e.* IL-1β, IL-6, IL-8, IL-10, IL-12p70 and TNF-α, as markers of systemic immune activation, and citrulline, a marker of functional enterocyte mass of the small bowel, were measured by multiplex immunoassay and high-pressure liquid chromatography (HPLC) fluorescence detection, respectively. The analyses and results were described previously.([Mujagic et al., 2016](#_ENREF_36)) In platelet poor plasma, systemic markers of serotonin metabolism were measured using high-performance liquid chromatography (HPLC), *i.e.* Serotonin (5-hydroxytryptamine, 5-HT) and 5-hydroxy indoleacetic acid (5-HIAA).([Thijssen et al., 2016](#_ENREF_48))

***Extended methods: faecal metabolic profiling***

Faecal metabolite concentrations were obtained using ^1^H-NMR spectroscopy, all faecal samples were defrosted and faecal water was extracted simultaneously, using standardized protocols.( [Gratton et al., 2016](#_ENREF_23)) The faecal sample aliquots were defrosted at room temperature, after which 1.0g faeces was transported into a 15mL Falcon tube (Eppendorf). 2mL of H_2_O (HPLC grade) was added and the sample was mixed using a multi-tube vortex mixer. Thereafter, the tubes were spun in a centrifuge at 10,000g for 10 minutes at 4°C, after which 2mL of the supernatant was transferred into a 2mL Eppendorf tube. The supernatant was stored at -80 °C until the second preparation step. For the second preparation step, all samples were defrosted at room temperature and were mixed using a vortex mixer. Then, the tubes were spun in a centrifuge at 18,000g for 10 minutes at 4°C. Thereafter, 400µL of faecal water supernatant is taken out into a new 1.5mL Eppendorf, and 250µL of buffer solution was added. The buffer solution contains 0.2M sodium phosphate, pH 7.4, 100% of deuterium oxide (D_2_O) for the field lock of the NMR spectrometer and 0.01% of sodium 3-(trimethylsilyl)-[2,2,3,3-^2^H_4_]-propionate (TSP) acting as a peak of reference at 0 ppm and 3mM NaN_3_ acting as bacteriostatic reagent.([Beckonert et al., 2007](#_ENREF_8)) This solution was vortexed and spun in a centrifuge for 2 minutes at 18,000g, thereafter 580µL of supernatant was transferred into an NMR tube with an outer diameter of 5mm for NMR analysis.

All NMR experiments were acquired using a spectrometer operating at 600.29 MHz for ^1^H and equipped with a 5mm, TCI, Z-gradient CryoProbe (Bruker BioSpin, Karlsruhe, Germany). The 1D ^1^H-NMR spectra were acquired using standard one‐dimensional pulse sequence (RD–gz–90°–t_1_–90°–t_mix_–gz–90°–ACQ), with saturation of the water resonance (noesygppr1d, Bruker nomenclature). The relaxation delay (RD) was set to 4s, short delay (t_1_) to 3μs and mixing time (t_mix_) to 10ms. Two magnetic field z‐gradients (gz) were applied for 1ms with a recovery delay of 200μs. The receiver gain was set to 90.5 and acquisition time (ACQ) to 2.73s for all experiments. Each spectrum was acquired using 4 dummy scans, and 32 scans that were collected into 64K data-points using a spectral width of 20 ppm. Free induction decays (FIDs) were multiplied by an exponential weighing function (corresponding to line broadening of 0.3Hz) prior to Fourier-transformation, referenced to TSP, manually phase-corrected, and baseline corrected using in-house software implemented in Matlab (R2014a, The Mathworks, Natick, MA, USA). Individual peaks were integrated and identified *a priori* using statistical methods([Posma et al., 2017](#_ENREF_38)), in-house databases and additional experiments([Garcia-Perez et al., 2020](#_ENREF_19)). Peaks that could not be identified were included in separate analyses, with the identified metabolites included in the main analyses.

***Extended methods: ^1^H-NMR data quality control***

The full resolution NMR were visualized using unsupervised analysis (Principal Component Analysis) by means of a pairs plot of the individual scores to identify outliers. Figure S1 shows the first 10 components from the model. Hotelling’s T^2^ statistic was calculated for these data and samples that mapped outside the 95% confidence bounds of the ellipsoid are indicated. The loadings were inspected to determine the reason for these outlying samples, the majority were identified as having high concentrations of polyethyleneglycol (PEG). The remaining samples were checked for any other structurFial variations and the baseline of all spectra was further corrected to ensure the data did not correlate with the peak width of the internal standard (Figure S2). The remaining data was used for analyses described in the main text methods and below. The identified metabolites in these data are given in Figure S3.

***Extended methods: data, statistical and bioinformatic analysis***

Support Vector Machine (SVM) classifiers (SVCs) ([Cortes and Vapnik, 1995](#_ENREF_15)) were utilized to develop a complete model for classifying the IBS and HC binary classes. The data was repeatedly split into training and test sets in an 80:20 ratio. All input data was standardized to unit variance by subtracting the mean from each value and dividing by the standard deviation. This results to a data distribution with standard deviation and variance that are equal to 1. The cost hyperparameter (C) behaves as a regularization parameter in all SVM models by trading off the correct classification of training samples against the maximization of the decision function’s margin. Grid search with 5-fold cross validation was used to find the optimal C values that maximized the accuracy for each linear model. The grid search evaluated 13 values starting from 0.001 and increasing on a log scale, and selected the value with the highest cross-validation accuracy. Hyperparameter tuning for the linear model was performed twice, before and after the feature selection.

Feature selection on the linear SVC models was performed by Recursive Feature Elimination (RFE) ([Guyon et al., 2002](#_ENREF_24)). RFE recursively considers smaller and smaller sets of features and the features with the lowest weights are removed from the input set. The process is recursively repeated until the optimal number of features is reached (maximizing prediction accuracy). The optimal number of features is pre-selected by performing RFE and cross validation. Features are then ranked based on the weight score and their class contribution is assigned based on the weight sign. The weights, calculated by the classifier, represent the vector coordinates (assigned to each feature) in relation to the hyperplane that separates the two classes. Their direction, positive or negative, indicates the predicted class.

The classifiers were trained 1,000 distinct times; where each time the training and test splits were done *ab initio*. Splits of the sets were tracked to ensure the same train-test split was assigned across the different models so as to allow for robust modelling. The main metric calculated for evaluating each model’s accuracy and effectiveness is the Area Under the ROC Curve (AUC) score. The ROC is a probability curve and AUC represents the degree of separability between the classes.

A combination of unsupervised learning methods was used to explore potential subgroupings within the IBS cohort. A three-step pipeline was developed by using dimensionality reduction, clustering, and statistical methods to assess the resulting groups. Kernel T-distributed Stochastic Neighbor Embedding (kt-SNE)([Gisbrecht A., 2015](#_ENREF_22)) is a non-linear dimensionality reduction method which fits a probability distribution on sets of the high-dimensional data. Similar points, based on Euclidean distances, are given a higher probability whereas dissimilar points a lower. A second probability distribution is calculated in the low-dimensional space and the algorithm aims to minimize the divergence of the two distributions with respect to the low-dimensional points. kt-SNE was used to reduce the more than 64 microbiome and metabolomic variables. These 64 variables were selected as the most important features by the previous SVC models. The 2-dimensional output coordinates from both methods were used as input in the clustering step.

K-means clustering is a partition algorithm which aims to separate n points in k clusters by assigning each point to the cluster with the nearest mean. This process is done a number of times so as to minimize the variance within each cluster by calculating the squared Euclidean distances of the points. To find the optimal number of clusters, the within-cluster-sum of squared errors (WSS) for 2 to 10 number of clusters was calculated. When plotting the WSS against the number of clusters we pick the point where WSS starts to flatten also visible as an elbow on the graph. This point is the value with the maximum second-order difference in WSS.

At the third step, a number of statistical tests were employed to assess the statistical significance of the resulting groups. The Kruskal-Wallis test by ranks was used to determine whether there are any statistically significant differences among the clusters (when k>2).  The Mann-Whitney U-test then determined the statistical significance between all sets of pairs of clusters. Both tests are non-parametric rank-sum tests that can be used in groups of the same or different sample size. The tests were applied on more than 200 phenotypic variables including demographic characteristics, lifestyle and dietary features, GI symptoms, Rome III subtypes, and others. This variety of clinical data was used to determine statistical significance between the clusters.  Finally, the Hommel correction – based on Simes’ test – was used to control the Family-Wise Error Rate (FWER) and correct for multiple testing across all the clinical data.

To assess the microbiome and metabolic features that play a role in the separation of the statistically significant groups, Support Vector Regression (SVR) was utilized for continuous variables and SVC for binary variables. Clinical data variables that showed significance between groups identified by unsupervised methods were included. All analyses were performed in Python (v3.7) using libraries scikit-learn (v0.23.2), pandas (v1.1.3), matplotlib (v3.3.2) and NumPy (v1.19.2).

Metabolic reaction networks were constructed based on the MetaboNetworks software ([Posma et al., 2014](#_ENREF_39)) (v2.3) and the KEGG database of biochemical reactions that occur in the human supra-organism. In addition to reactions mediated by human enzyme-coding genes, here we included enzymatic reactions from all microbial species in KEGG that belong to the families that were identified from the microbiome analyses here. The constructed database considers that two metabolites are associated with each other if a biochemical reaction entry in KEGG indicates that they are a main reactant pair and the enzyme involved is linked to a human or microbial gene. We visualize the association of microbiota with each reaction by looking at individual species in each family and which genes have been mapped to specific enzymes involved in each reaction. KEGG incorporates this information from UniProt into its database and combines them with other information. We visualize these as part of the full metabolic network (Figure S4) and highlight specific cases where at least one metabolite is part of a reaction, either as substrate or product, that can be catalysed by a microbial enzyme. Metabolites that are significantly associated with IBS or HC but not involved in any reaction that is mediated by the IBS-associated microbiota, are shown in the full network.

***Supplementary tables***

| **Biomarkers measured in sub-populations** | | |
| --- | --- | --- |
| **Fecal biomarkers** | **170 IBS** | **103 HC** |
| Calprotectin (ug/g) | 63.5 ± 87.8* | 36.1 ± 49.9 |
| Human beta defensin 2 (ng/g) | 41.3 ± 43.5* | 61.3 ± 83.5 |
| Chromogranin A (nmol/g) | 38.0 ± 45.8*** | 22.8 ± 29.2 |
| **Cytokines measured in plasma** | **140 IBS** | **102 HC** |
| Interleukin 1-beta (ug/L) | 0.48 ± 0.65** | 1.03 ± 2.6 |
| Interleukin 6 (ug/L) | 1.64 ± 10.9 | 2.88 ± 7.9 |
| Interleukin 10 (ug/L) | 0.94 ± 0.60 | 0.89 ± 0.58 |
| Interleukin 12 p70 (ug/L) | 2.34 ± 2.20 | 1.93 ± 4.22 |
| Interleukin 10 / 12 ratio | 2.56 ± 5.70** | 4.33 ± 6.83 |
| TNF-alfa (ug/L) | 1.74 ± 2.46 | 2.11 ± 8.87 |
| **Serotonin markers in platelet poor plasma** | **104 IBS** | **84 HC** |
| 5-hydroxytryptamine (5-HT, serotonin; nmol/L) | 34.2 ± 29.5* | 40.4 ± 51.7 |
| 5-hydroxyindoleacetic acid (5-HIAA; nmol/L) | 128 ± 107*** | 207 ± 146 |
| 5-HIAA/5-HT ratio | 7.46 ± 10.2*** | 13.5 ± 29.2 |
| **Visceral hypersensitivity, by rectal barostat** | **109 IBS** | **68 HC** |
| Pain VAS score at balloon pressure of 26mmHg | 27.2 ± 29.8*** | 6.4 ± 13.1 |
| **Small intestinal barrier marker in plasma** | **140 IBS** | **102 HC** |
| Citrulline (umol/L) | 42.4 ± 12.5 | 40.3 ± 10.6 |
| **Intestinal permeability by multi-sugar test** | **48 IBS** | **54 HC** |
| Lactulose/rhamnose ratio in 0-5h urine (indicating small intestinal permeability) | 0.019 ± 0.015 | 0.023 ± 0.027 |
| Sucralose/erythritol ratio in 5-24 urine  (indicating colonic permeability) | 0.021 ± 0.048 | 0.012 ± 0.009 |

**Table S1: Biomarkers measured IBS patients versus HC, all presented as mean ± SD**

Data on presented markers has been published previously.([*11*](#_ENREF_11)*,* [*14-16*](#_ENREF_14)) Differences tested with independent samples t-test; **P<*0.05; ***P<*0.01; ****P*<0.001 vs. HC.

***Supplementary figures***


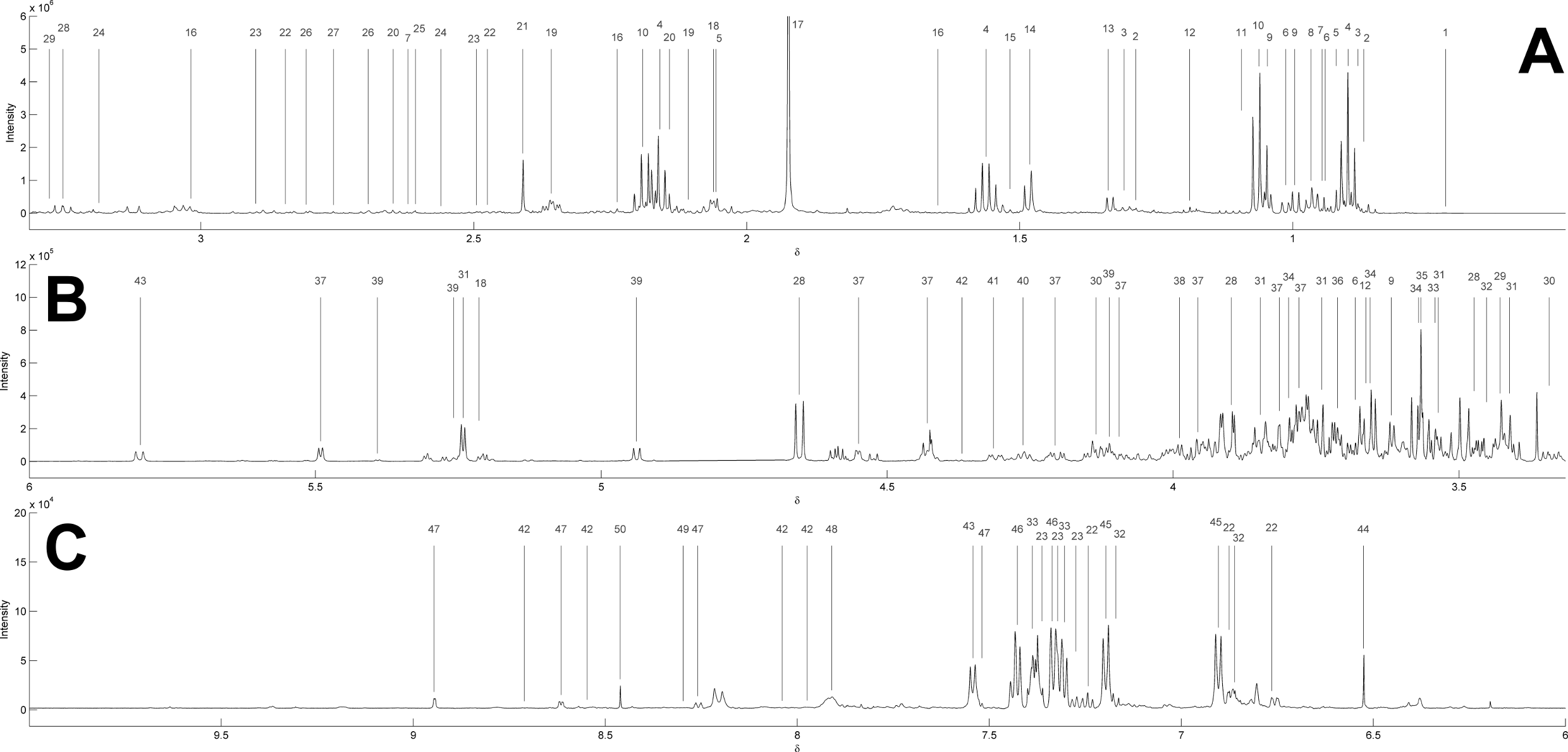


**Figure S1. Representative partial 600 MHz ^1^H-NMR spectra of human fecal water samples (mean of all data) showing assignments of the identified metabolic signals.** Full 600 MHz ^1^H-NMR spectrum is shown in 3 panels, (A) δ 0.5–3.3 ppm, (B) δ 3.3–6.0 ppm and (C) δ 6.0–10.0 ppm. Key: 1 – bile salts, 2 – caproate, 3 – valerate, 4 – butyrate, 5 – isovalerate, 6 – isoleucine, 7 – 2-oxoisocaproate, 8 – leucine, 9 – valine, 10 – propionate, 11 – methylsuccinate, 12 – ethanol, 13 – lactate, 14 – alanine, 15 – 2-methylbutyrate, 16 – 5-aminovalerate, 17 – acetate, 18 – *N*-acetylglucosamine, 19 – glutamate, 20 – methionine, 21 – succinate, 22 – 3-hydroxyphenylpropionate, 23 – 3-Phenylpropionate, 24 – Beta-alanine, 25 – Methylamine, 26 – aspartate, 27 – 2-methylproline, 28 – beta-glucose, 29 – taurine, 30 – proline, 31 – alpha-glucose, 32 – 4-hydroxyphenylacetate, 33 – phenylacetate, 34 – glycerol, 35 – glycine, 36 – polyethyleneglycol (PEG), 37 – sucralose, 38 – serine, 39 – ribose, 40 – threonine, 41 – malate, 42 – homarine, 43 – uracil, 44 – fumarate, 45 – tyrosine, 46 – phenylalanine, 47 – nicotinate, 48 – xanthine, 49 – 3-methylhistidine, 50 – formate.


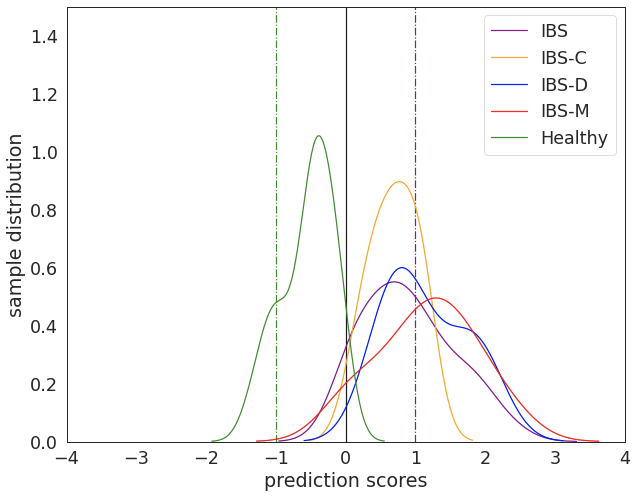


**Figure S2. Combined linear recursive feature elimination (RFE) for IBS versus HC for the multiomics analysis in which IBS is split into 3 clinical subtypes.** The purple (IBS) and green (HC) lines are the same as in Figure 3A. The yellow (IBS-C), blue (IBS-D) and red (IBS-M) lines are the normalized kernel density estimates of the 3 subtypes. A comparison between the 3 subtypes based on pairwise Mann-Whitney U-tests did not indicate there are differences in the predicted scores between the 3 subtypes.


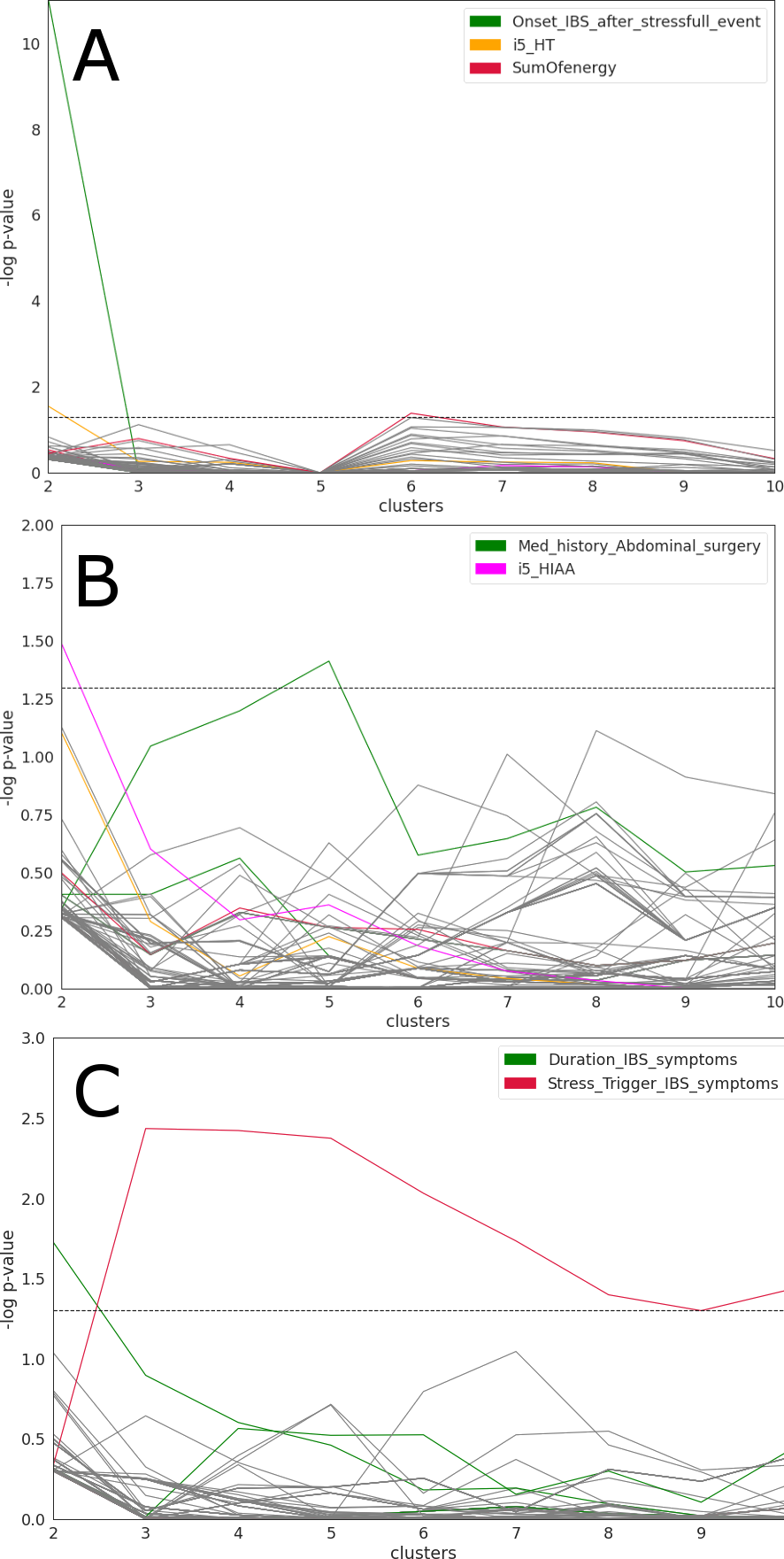


**Figure S3. Subgroup analyses of clusters obtained from kt-SNE analyses and testing these for differences in clinical and meta data.** (A) For clusters obtained from the family-level microbiome data only. (B) For clusters obtained from metabolite data only. (C) For clusters obtained from the integration of family-level taxonomic data with fecal metabolites. The vertical axis indicates statistical significance after multiple testing corrections of the *P*-values (*P*_adjusted_≤0.05) represented on log_10_ scale.


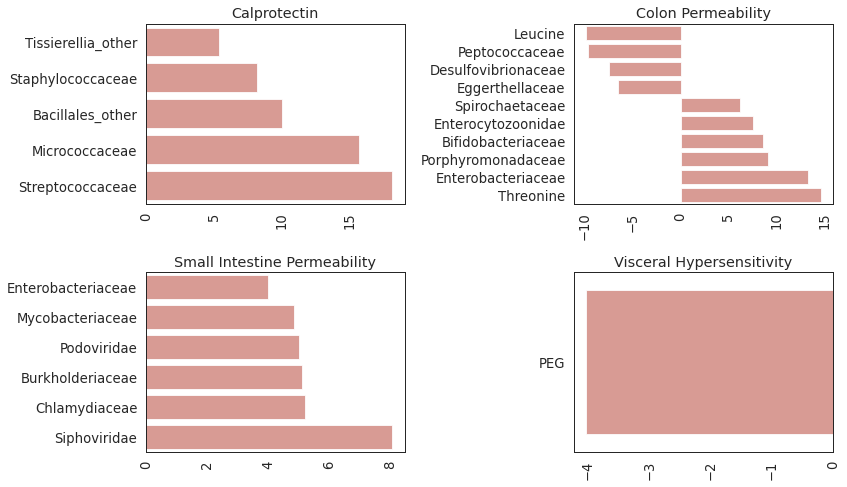


**Figure S4. Important features for SVR models of specific clinical markers. (A)** Microbiota families associated with calprotectin (no metabolites are associated but were included in model). **(B)** Fecal metabolites and microbiota families associated with colon permeability. **(C)** Microbiota families associated with small intestine permeability (no metabolites are associated but were included in model). **(D)** Polyethylene glycol is the only feature (among metabolites and microbiota families) associated with visceral hypersensitivity, but this is a drug metabolite, a laxative.


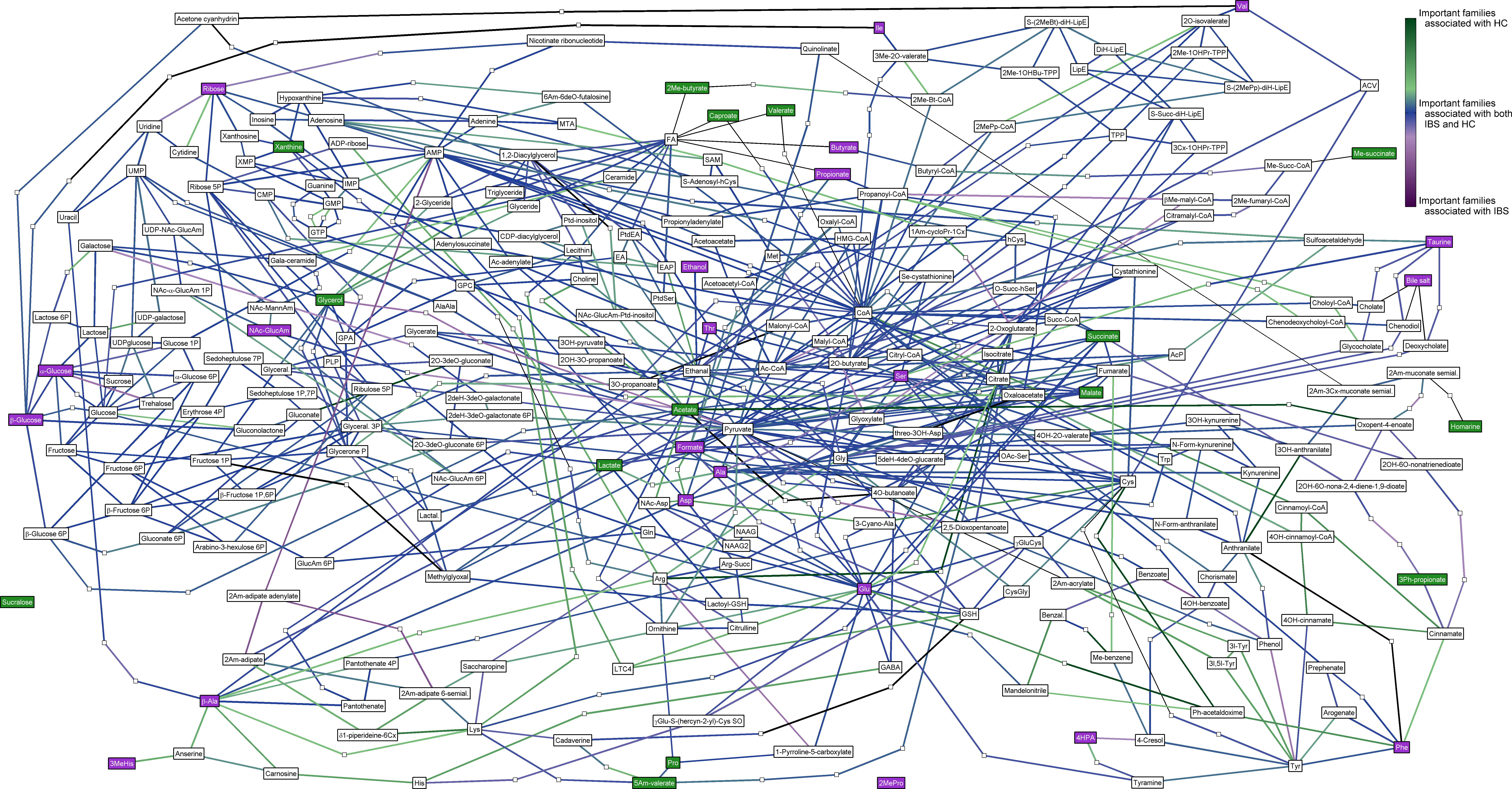


**Figure S5. Metabolic reaction network integrated with microbiome information.** Each nodes (box) indicates a metabolite and each edge (line connecting two nodes) indicates these metabolites are involved in an enzymatic reaction. The network includes both host (*Homo sapiens*) and microbial (based on identified families) mediated reactions. Green nodes indicate metabolites associated with healthy controls (HC) and purple nodes indicate metabolites associated with IBS. For each edge the relative proportion of healthy- versus IBS-associated bacterial families that have the required enzyme (that mediate reactions involving each metabolite pair) was calculated. The edge color indicates the relative number of associated families, with green indicating the bacteria with this enzyme are mostly associated with HCs, purple indicates the families are mostly associated with IBS, and blue indicating the proportions are relatively equal. Black edges indicate reactions that are known to occur, but not associated with any of the associated families with HC or IBS.


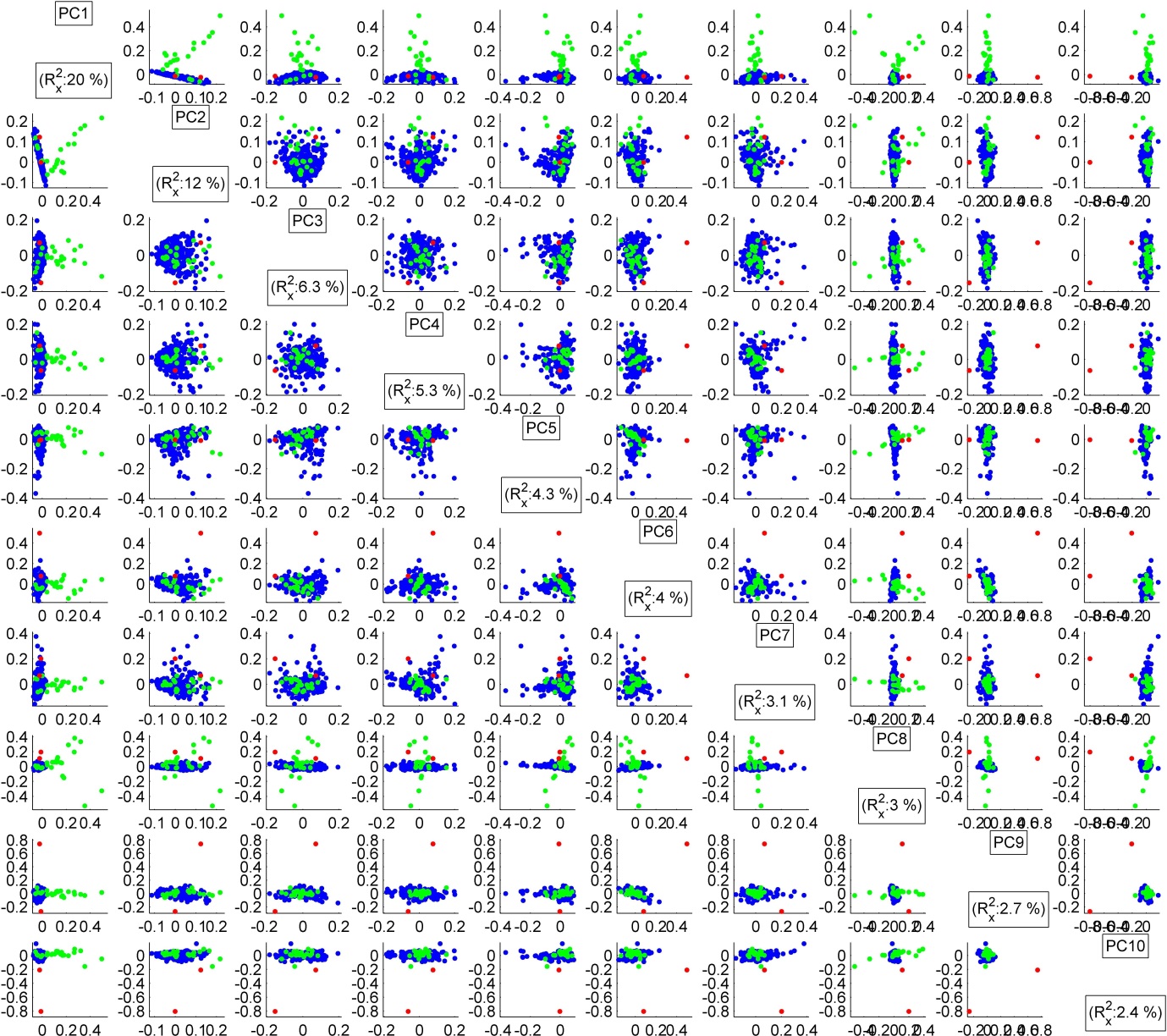


**Figure S6. Principal Component Analysis (PCA) pairs plot (first 10 components) visualizing outlier removal (n=23 with polyethyleneglycol (PEG) in green, n=2 others in red) based on Hotelling’s T^2^ statistic.**


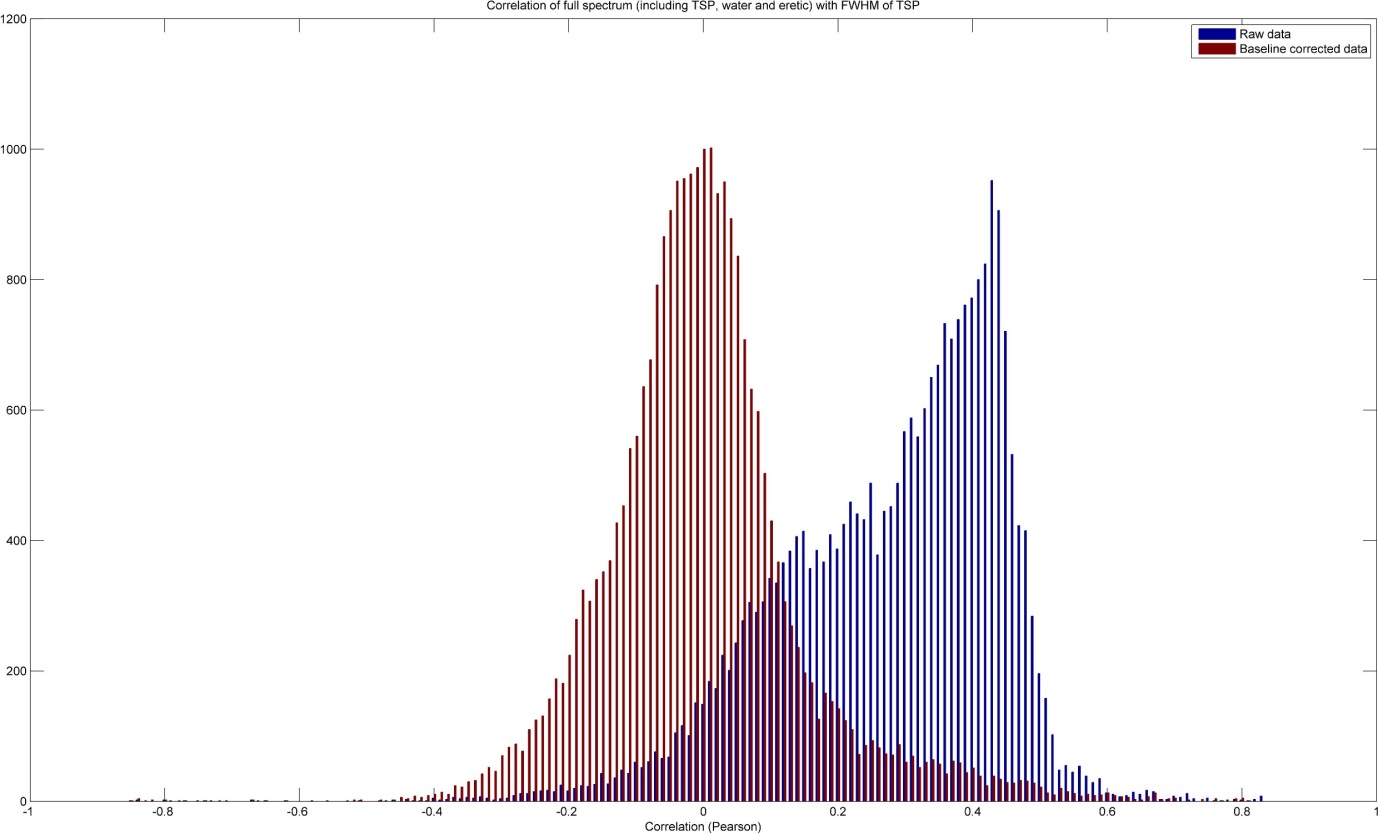


**Figure S7. Baseline correction of the ^1^H-NMR data indicates an absence of influence from spectral linewidth on spectra after correction as evident by the normal distribution.** Data show the correlation of ^1^H-NMR variables with the full-width at half-maximum (FWHM) of the chemical reference standard TSP, blue = before correction and red = after correction.


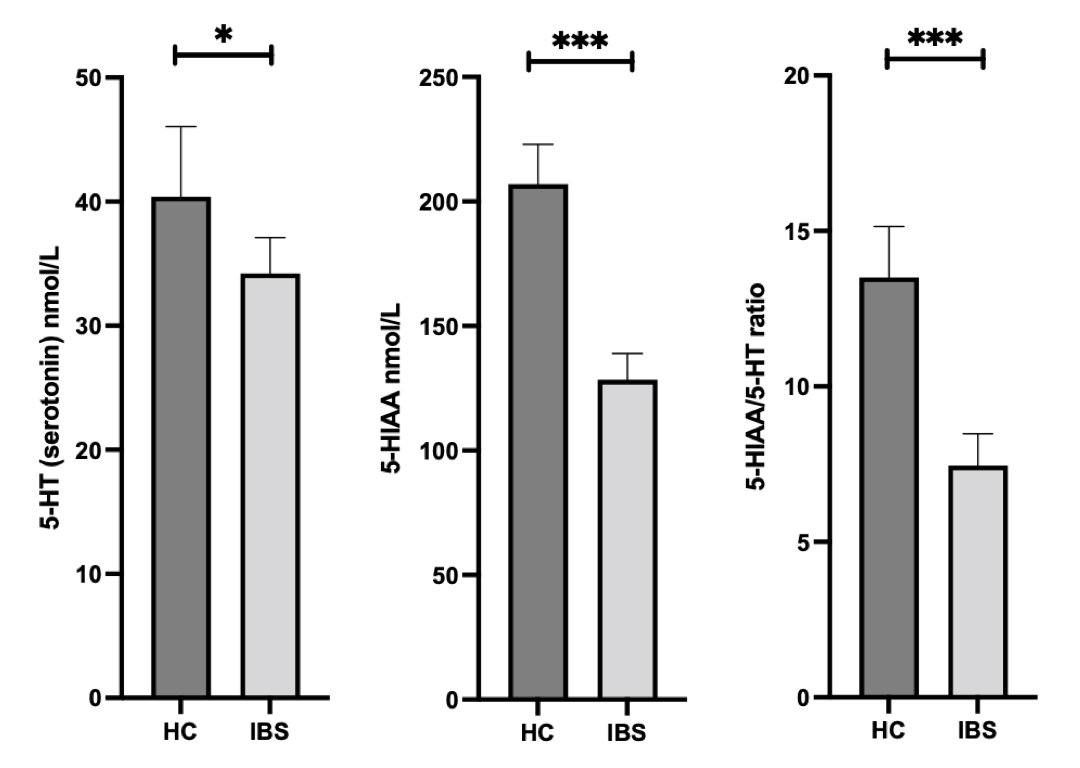


**Figure S8.** Boxplots of platelet poor plasma 5-HT, 5-HIAA and ratio of both (means (SEM)) for IBS and HC. Differences tested with independent samples t-test; **P<*0.05; ****P*<0.001 vs. HC.
